# Sex-dependent associations of low birth weight and suicidal ideation in adulthood: a community-based cohort study

**DOI:** 10.1038/s41598-020-69961-5

**Published:** 2020-07-31

**Authors:** Mareike Ernst, Iris Reiner, Achim Fieß, Ana N. Tibubos, Andreas Schulz, Juliane Burghardt, Eva M. Klein, Elmar Brähler, Philipp S. Wild, Thomas Münzel, Jochem König, Karl J. Lackner, Norbert Pfeiffer, Matthias Michal, Jörg Wiltink, Manfred E. Beutel

**Affiliations:** 1 Department of Psychosomatic Medicine and Psychotherapy, University Medical Center of the Johannes Gutenberg-University Mainz, Untere Zahlbacher Str. 8, 55131 Mainz, Germany; 2 Department of Ophthalmology, University Medical Center of the Johannes Gutenberg-University Mainz, Langenbeckstr. 1, 55131 Mainz, Germany; 3 Preventive Cardiology and Preventive Medicine - Center for Cardiology, University Medical Center of the Johannes Gutenberg-University Mainz, Langenbeckstr. 1, 55131 Mainz, Germany; 4 Center for Thrombosis and Hemostasis (CTH), University Medical Center of the Johannes Gutenberg-University Mainz, Langenbeckstr. 1, 55131 Mainz, Germany; 5 German Center for Cardiovascular Research (DZHK), Partner Site Rhine-Main, Langenbeckstr. 1, 55131 Mainz, Germany; 6 Center for Cardiology - Cardiology I, University Medical Center of the Johannes Gutenberg-University Mainz, Langenbeckstr. 1, 55131 Mainz, Germany; 7 Institute of Medical Biostatistics, Epidemiology and Informatics, University Medical Center of the Johannes Gutenberg-University Mainz, Obere Zahlbacher Str. 69, 55131 Mainz, Germany; 8 Institute of Clinical Chemistry and Laboratory Medicine, University Medical Center of the Johannes Gutenberg-University Mainz, Langenbeckstr. 1, 55131 Mainz, Germany

**Keywords:** Psychology, Depression, Epidemiology, Risk factors

## Abstract

Low birth weight (LBW; < 2,500 g) has been identified as a risk factor for adverse mental health outcomes over the life span. However, little is known about the association of LBW and suicidal ideation in middle and late adulthood. We investigated *N* = 8,278 participants of a representative community cohort: 3,849 men (46.5%) and 4,429 women (53.5%) (35–74 years of age). We assessed standardized measures of mental distress, sociodemographics, health behavior, and somatic factors (based on an extensive medical assessment). Controlling for these confounders, we examined the relationship of birth weight and suicidal ideation in logistic regression models. As men and women differ with regard to their susceptibility to suicidal ideation and behavior, we tested sex-dependent effects. LBW was reported by 458 participants (5.5%). In men, LBW was associated with a higher likelihood of reporting suicidal ideation (OR 2.92, 95% CI 1.58–5.12). In women, there was no such relationship. The findings underscore the interrelatedness of the physical and psychological domain, the role of early adversity in suicidal ideation, and they identify a vulnerable group whose numbers are expected to grow. They also indicate other risk factors for suicidal ideation in the community (mental distress, lack of social support, and health risk behavior).

## Introduction

Suicide is a major mental health problem which annually accounts for more than 150,000 deaths in Europe and 800,000 deaths worldwide. In many countries, rates of death by suicide are currently on the rise in numerous populations^1^. Thus, paramount aims of research are the identification of risk factors and the advancement of explanatory models in order to better recognize those at risk and to provide evidence-based prevention and intervention efforts. This is still a difficult task^2^ as the etiology of suicidal ideation and behavior is complex. Diverse biological, social, and psychological variables play a role^3^, and some established risk factors (such as higher age, male sex, and mental disorders, including drug/alcohol abuse disorders) are very common in the community^2^. As there is an urgent need for early diagnosis and treatment of individuals at risk, a promising approach could be to consider variables which occur at an early stage of life and to investigate them in an interdisciplinary manner^4^.

In fact, a large body of evidence suggests that early adverse experiences shape mental health outcomes over the life span. It is generally acknowledged that adversity can start as early as in the womb and that the offspring’s developmental trajectories can be substantially influenced by perinatal factors. However, only few studies to date have investigated suicidal ideation and behavior later in life in conjunction with respective variables such as low birth weight^5–8^.

Low birth weight (LBW), defined as below 2,500 g by the World Health Organisation^9^, has been a subject of clinical and epidemiological investigation for a long time. Before the introduction of neonatal intensive care and further improvements in perinatal management in the 1960s, LBW strongly affected infant mortality and childhood morbidity rates. As the rate of LBW infants has been growing during the recent century and their survival rates are also on the rise, an increasing number of individuals with LBW will reach adulthood and old age. Causes of LBW are either a preterm or short gestation period, retarded intrauterine growth or a combination of both^10^. LBW may also indicate prenatal stress^11^, a risk factor for behavioral and mental health problems later in life^12^. To date, follow-up studies have shown associations of LBW with delay in achieving developmental and psychosocial milestones including partnership and parenthood^13^, lower educational attainment and income^14^, and negative physical consequences such as cardiovascular disease and diabetes^15,16^. Further, LBW babies were at an increased risk of depression and anxiety in childhood^17,18^, behavioral problems and ADHD symptoms in adolescence^19^, and mental disorders in early adulthood^7,20^. Evidence also suggests an association with depression in middle to late adulthood^10, 21,22^.

Little is known concerning the relevance of LBW for suicidality. Although large cohort studies found an increased risk of suicide for those born with LBW, one study only investigated suicide attempts and suicide deaths which took place before the age of 27 years^6^. Another study used a sample with a mean age of 25 years^5^. It is therefore not clear whether the link of LBW and suicidality extends into middle and late adulthood. However, in high-income countries, numbers of suicide deaths are highest in the middle-aged and the elderly^1^. Thus, we need to learn more about possible risk factors for this age group as well.

From this evidence arises a need to investigate the links of perinatal factors such as LBW with suicidality in middle to late adulthood. Importantly, previous work outlined above has highlighted the associations of LBW with somatic aspects. A comprehensive investigation of the relevance of LBW for suicidality, a specific form of mental distress, should thus take account of the biopsychosocial context: By exploring the contribution of sociodemographic data, somatic illnesses, and also measures of health behavior, which may reflect the impact of poor emotion regulation strategies on the body (e.g. in the case of smoking)^23^. On the same note, striking sex differences with respect to suicidal ideation and suicidal actions call for sex-sensitive methods of inquiry, namely the preponderance of female suicide attempters compared to male deaths by suicide. Studies have also suggested that outcomes of LBW adults might differ depending on sex. However, there is disagreement whether men^24^ or women^25^ constitute the more vulnerable group.

Based on a comprehensive data set of a large, population-based adult sample (*N* = 15,010; ages 35–74 years) our aims were to determine:

1. whether low birth weight is associated with suicidal ideation in middle and late adulthood (while controlling for other factors shaping mental health such as sociodemographic variables, somatic illnesses, and health behavior).
2. whether low birth weight is related to suicidal ideation in men and women in different ways.

## Method

### Procedure and study sample

The Gutenberg Health Study (GHS) is a population-based, prospective, observational single-center cohort study in the Rhine-Main-Region, Germany. Its procedure is described in more detail elsewhere^26,27^. The project’s primary aim is to analyze (risk) factors that are relevant to the health of the population. Thus, it yields detailed information about the population’s health condition. The study protocol and documents were approved by the ethics committee of the Medical Chamber of Rhineland-Palatinate and the local data safety commissioner. All investigations were conducted in line with the Declaration of Helsinki and principles outlined in recommendations for Good Clinical Practice and Good Epidemiological Practice. Before inclusion, informed consent was obtained from all participants. The sample was drawn randomly from the local registry in the city of Mainz and the district of Mainz-Bingen, stratified 1:1 for sex and residence and in equal strata for decades of age. Inclusion criterion was age between 35 and 74 years. Exclusion criteria pertained to insufficient knowledge of German and psychological or physical impairment which was so grave that individuals were not able to travel to the study center. At baseline (between 2007 and 2012), 15,010 participants were examined. Data used in the present analyses was drawn from this baseline investigation.

### Materials and methods

During the extensive 5-h examination in the study center, cardiovascular risk factors and other clinical variables were assessed, complemented by a computer-assisted personal interview, laboratory examinations from venous blood samples, blood pressure, and anthropometric measurements. All examinations were performed by certified medical technical assistants and followed standard operating procedures.

Reported sociodemographic variables were assessed via self-report: sex, age in years, living with a partner (no/yes), and socioeconomic status (SES).

SES was defined according to Lampert et al.^28^. The index ranges from 3 (lowest socioeconomic status) to 21 (highest socioeconomic status) and combines information on education, occupation, and income with equal weights.

Participants were asked to review personal birth records or family albums for recorded birth weight. All participants with self-reported birth weight (BW) were included. A non-responder analysis is provided below. Participants reporting < 2,500 g were assigned to the LBW group and those reporting 2,500–4,000 g were defined as having had normal BW. BWs > 4,000 g were defined as high. In line with previously established study procedures, participants with BWs < 1,000 g and > 6,000 g were excluded as these self-report data were deemed unreliable^29^. The World Health Organization also recommends using a 1,000-g threshold for international comparisons of perinatal mortality rates^9^.

#### Mental distress measures

Suicidal ideation was measured using the item “Over the past two weeks, how often have you been bothered by thoughts that you would be better off dead or of hurting yourself in some way?” from the Patient Health Questionnaire’s (PHQ) depression module PHQ-9. The PHQ-9 quantifies the frequency of being bothered by 9 diagnostic criteria of Major Depression and is a widely used questionnaire with good internal consistency (in our sample, Cronbach’s *α* was 0.80). For each item, occurrence over the past two weeks was rated from 0 = not at all, 1 = several days, and 2 = more than half the days to 3 = nearly every day. The presence of clinically relevant symptom burden has been defined by a cut-off-score ≥ 10 for the PHQ-9^30^ as well as for the PHQ-8 (i.e. excluding the suicidal ideation item)^31^. In the following, references to “(relevant) depression symptoms” within the context of the present study relate to the PHQ-8 scores.

To assess symptoms of generalized anxiety disorder, we used the GAD-2, the established two-item short form of the Generalized Anxiety Disorder-7 scale^32,33^. Responses range from 0 = not at all to 3 = nearly every day (addressing being bothered by symptoms within the past two weeks). The items are “Feeling nervous, anxious or on edge” and “Not being able to stop or control worrying”. For the GAD-2, a sum score of 3 or higher has previously indicated generalized anxiety with good sensitivity (86%) and specificity (83%)^34^.

The German version of the Mini-Social Phobia Inventory (Mini-Spin) was used to detect social anxiety symptoms. Three items describe behavior of avoidance and fear of embarrassment (e.g. “I avoid activities in which I am the center of attention”) on a 5-point Likert-scale ranging from 0 = not at all to 4 = extremely. A cut-off score of 6 has been found to separate individuals with generalized social anxiety disorder and controls with good sensitivity (89%) and specificity (90%)^35^.

Panic disorder was screened for with the brief PHQ panic module. The screening question was “In the last 4 weeks, have you had an anxiety attack—suddenly feeling fear or panic?” followed by 4 specific questions. All items have a binary response format (no/yes). Caseness was defined as at least two of the four PHQ panic questions answered with “yes”^36^.

#### Health behavior measures

Smoking was dichotomized into non-smokers (never or ex-smoker) and smokers (occasional and regular smoker). Obesity was defined as a body mass index ≥ 30 kg/m^2^. Alcohol consumption was measured in gram per day,alcohol abuse was defined as daily consumption ≥ 60 mg for men and ≥ 40 mg for women. Physical activity was inquired with the Short Questionnaire to Assess Health-Enhancing Physical Activity (SQUASH)^37^. The SQUASH captures active sports, commuting, leisure time, household, work and school activities. Sleeping, lying, sitting and standing were classified as inactivity.

#### Somatic illnesses

Self-reported myocardial infarction (MI), congestive heart failure (CHF), stroke, coronary artery disease (CAD), and peripheral arterial disease (PAD) were summarized as cardiovascular disease (CVD). Coronary artery disease was assessed by the question: “Were you diagnosed with a stenosis of your coronary vessels?”. Cancer and medicated obstructive pulmonary disease (COPD/asthma) were also assessed via self-report. Diabetes was defined in individuals with a definite diagnosis of diabetes by a physician or a blood glucose level of ≥ 126 mg/dl in the baseline examination after an overnight fast of at least 8 h or a blood glucose level of > 200 mg/dl after a fasting period of 8 h.

### Statistical analyses

Descriptive analyses were performed as absolute frequencies and proportions for categorical data, means and standard deviations for continuous variables and medians with interquartile ranges if not fulfilling normal distribution. Inference tests between groups (of those with and without LBW; and of responders and non-responders) were calculated with t-tests or χ^2^ tests, where appropriate. In the following, “presence of suicidal ideation” means that participants reported suicidal ideation at least on several days (i.e. response categories 1–3 of the respective PHQ-9 item). We tested the association of LBW with suicidal ideation in two steps: First, we tested a regression model for the whole sample which included the predictors LBW (vs. normal birth weight), the interaction term of LBW (vs. normal birth weight) with sex, and predictors of the domains demographics, health behavior, somatic illness, and psychological comorbidities. Then, we calculated sex-specific models retaining the predictor LBW (vs. normal birth weight). All regression analyses controlled for the presence of high (> 4,000 g) vs. normal BW and for an interaction term of high BW with sex as there is evidence for associations of high BW with health outcomes^38^ and potential sex differences with regard to this relationship^39^. P-values are two-sided. They should be regarded as continuous parameters that reflect the level of statistical evidence and are therefore reported exactly. Univariate comparisons are supplemented by effect sizes (Cohen’s *d*)^40^. All analyses were carried out using R version 3.3.1 (https://www.R-project.org). In the Tables, statistically significant results are printed in bold.

## Results

### Sample characteristics

In total, 8,369 participants (56% of the total sample) reported their BW. Forty-five individuals were excluded due to reports of BW below 1,000 g (*n* = 7) or above 6,000 g (*n* = 38). Further, from *n* = 91, mental distress measures were not available. Thus, 8,278 participants constitute the analysis set. Table 1 gives an overview of the participants with and without LBW. With 453 cases, 5.5% of participants reported having been born with LBW. Among those with LBW were more women. Normal BW was reported by 6,778 individuals (81.9% of the total sample). High BW was reported by 1,047 participants (12.6% of the total sample). Among those individuals were more men. Overall, mean age was 51.5 (*SD* = 10.6) years. Participants with LBW were older and had a lower SES than participants with normal/high BW. Further univariate group differences pertained to higher rates of mental distress, except depression symptoms, in participants with LBW.

**Table 1:** Sample characteristics of the Gutenberg Health study participants, stratified by birth weight.

	All (*N* = 8,278)	BW above 2,500 g (*N* = 7,825, 94.5%)	Low BW^a^ (*N* = 453, 5.5%)	*p*	*d*
**Demographics**					
Birth weight (g)	3,409 (656)	3,491 (571)	2,000 (384)	** < 0.001**	3.06
Sex (% women)	53.5 (4,429)	52.7 (4,122)	67.8 (307)	** < 0.001**	0.14
Age (years)	51.5 (10.6)	51.4 (10.6)	53.2 (11.1)	** < 0.001**	0.17
Living with partner (%)	81.4 (6,736)	81.4 (6,366)	81.7 (370)	0.90	0.00
Socioeconomic Status	13.63 (4.28)	13.66 (4.28)	13.12 (4.16)	**0.008**	0.13
**Health behavior**					
Smoking (%)	20.4 (1,690)	20.5 (1,604)	19.0 (86)	0.47	0.02
Obesity (%)	23.7 (1,958)	23.6 (1,845)	24.9 (113)	0.50	0.01
Active sports (%)	53.2 (4,404)	53.2 (4,163)	53.2 (241)	> 0.99	0.00
Alcohol (gram/day)	4.71 (0/15.71)	5.03 (0/16.15)	2.51 (0/14.14)	**0.02**	0.06
**Somatic illness**					
Diabetes (%)	6.4 (526)	6.3 (493)	7.3 (33)	0.43	0.02
Cardiovascular disease (%)	8.3 (691)	8.3 (651)	8.8 (40)	0.66	0.01
Cancer (%)	7.7 (638)	7.6 (597)	9.1 (41)	0.28	0.02
COPD/asthma (%)	4.8 (395)	4.7 (368)	6.0 (27)	0.21	0.03
**Mental distress (%)**					
Suicidal ideation	6.9 (568)	6.6 (514)	11.9 (54)	** < 0.001**	0.10
Depression symptoms	7.6 (632)	7.5 (590)	9.3 (42)	0.17	0.03
Anxiety symptoms	6.8 (560)	6.6 (517)	9.5 (43)	**0.02**	0.05
Social phobia symptoms	7.9 (648)	7.7 (601)	10.4 (47)	0.05	0.05
Panic symptoms	4.8 (391)	4.6 (352)	8.9 (39)	** < 0.001**	0.09

Results reported as % (*n*), mean (standard deviation) or median (1st quartile/3rd quartile). Inference tests between groups (normal/low birth weight) were calculated with t-tests or Chi^2^ tests.

BW, birth weight; COPD, chronic obstructive pulmonary disease.

^a^According to the World Health Organization, low birth weight was defined as below 2,500 g.

As the present study aims to examine possible sex differences with respect to the association of LBW with suicidal ideation in adulthood, sample characteristics are also presented stratified by sex (Table 2). Women with LBW were older and reported lower SES compared to women with higher BW. Men with LBW more often reported anxiety, panic symptoms, and suicidal ideation. Among women, differences with regard to suicidal ideation between those with LBW and higher BW (10.4% vs. 7.7%) fell short of statistical significance. There was no statistically significant difference regarding other kinds of mental distress either.

**Table 2:** Sample characteristics, stratified by birth weight and sex.

	Men					Women				
	All (*N* = 3,849)	BW > 2,500 g (*N* = 3,703)	Low BW^a^ (*N* = 146)	*p*	*d*	All (*N* = 4,429)	BW > 2,500 g (*N* = 4,122)	Low BW^a^ (*N* = 307)	*p*	*d*
**Demographics**										
Birth weight (g)	3,550 (659)	3,611 (590)	2,005 (399)	** < 0.001**	3.19	3,286 (629)	3,382 (530)	1,998 (378)	** < 0.001**	3.01
Age (years)	51.5 (10.6)	51.5 (10.5)	51.9 (11.2)	0.66	0.04	51.5 (10.6)	51.3 (10.6)	53.9 (11.1)	** < 0.001**	0.24
Living with partner (%)	84.1 (3,238)	84.2 (3,118)	82.2 (120)	0.49	0.02	79.0 (3,498)	78.8 (3,248)	81.4 (250)	0.31	0.03
Socioeconomic status	14.45 (4.34)	14.44 (4.34)	14.63 (4.29)	0.61	0.04	12.91 (4.09)	12.95 (4.10)	12.40 (3.91)	**0.02**	0.14
**Health behavior**										
Smoking (%)	21.3 (821)	21.3 (788)	22.6 (33)	0.68	0.01	19.6 (869)	19.8 (816)	17.3 (53)	0.33	0.02
Obesity (%)	25.5 (982)	25.5 (944)	26.0 (38)	0.92	0.00	22.0 (976)	21.9 (901)	24.4 (75)	0.32	0.03
Active sports (%)	50.8 (1956)	50.8 (1,881)	51.4 (75)	.93	.00	55.3 (2,448)	55.4 (2,282)	54.1 (166)	0.68	0.01
Alcohol (gram/day)	8.44 (0/22.63)	8.44 (0/22.63)	7.12 (0/22.51)	0.88	0.04	0 (0/9.43)	0 (0/9.43)	0 (0/8.80)	0.30	0.02
**Somatic illness**										
Diabetes (%)	8.0 (309)	7.9 (293)	11.0 (16)	0.21	.04	4.9 (217)	4.9 (200)	5.5 (17)	0.58	0.02
Cardiovascular disease (%)	11.0 (423)	11.0 (409)	9.6 (14)	0.69	0.02	6.1 (268)	5.9 (242)	8.5 (26)	0.08	0.06
Cancer (%)	6.4 (245)	6.3 (233)	8.2 (12)	0.38	0.03	8.9 (393)	8.8 (364)	9.4 (29)	0.68	0.01
COPD/asthma (%)	3.7 (144)	3.7 (137)	4.8 (7)	0.50	0.02	5.7 (251)	5.6 (231)	6.5 (20)	0.52	0.02
**Mental distress (%)**										
Suicidal ideation	5.8 (221)	5.4 (199)	15.1 (22)	** < 0.001**	0.16	7.9 (347)	7.7 (315)	10.4 (32)	0.09	0.05
Depression symptoms	5.7 (220)	5.6 (208)	8.2 (12)	0.20	0.04	9.3 (412)	9.3 (382)	9.8 (30)	0.76	0.01
Anxiety symptoms	5.1 (196)	5.0 (184)	8.2 (12)	0.08	0.06	8.2 (364)	8.1 (333)	10.1 (31)	0.23	0.04
Social phobia symptoms	6.2 (237)	6.1 (224)	8.9 (13)	0.16	0.05	9.3 (411)	9.2 (377)	11.1 (34)	0.26	0.03
Panic symptoms	3.3 (126)	3.1 (112)	9.8 (14)	** < 0.001**	.14	6.2 (265)	6.0 (240)	8.5 (25)	0.10	0.05

Results reported as % (*n*), mean (standard deviation) or median (1st quartile/3rd quartile). Inference tests between groups (normal/low birth weight) were calculated with t-tests or Chi^2^ tests.

BW, birth weight; COPD, chronic obstructive pulmonary disease.

^a^According to the World Health Organization, low BW was defined as below 2,500 g.

### Associations of suicidal ideation with LBW and other predictors

Table 3 shows the results of the multivariate logistic regression analyses exploring the risk to report suicidal ideation. It included all participants for whom data was available with respect to the listed potential predictors. As delineated above, we tested the statistical effects of LBW as well as high BW and those categories’ interactions with sex. Additional demographic factors comprised sex, age (we tested the statistical effect per 5 years of age), living together with a partner, and SES. Health behavioral factors were obesity, smoking, active sports, and alcohol abuse. Regarding the effects of somatic illnesses, the model included diabetes, CVD, cancer, and COPD.

**Table 3:** Logistic regression of suicidal ideation on birth weight categories controlling for health behaviour, somatic illnesses, and psychological comorbidities (*N* = 8,000; *N* = 536 events).

	OR	95% CI (L, U)	*p*
**Demographics**			
Low birth weight (< 2,500 g) vs. normal birth weight	2.84	1.56, 4.92	** < 0.001**
High birth weight (> 4,000 g) vs. normal birth weight	0.64	0.39, 1.00	0.06
Low birth weight (< 2,500 g) vs. normal birth weight * Sex	0.48	0.23, 1.01	**0.047**
High birth weight (> 4,000 g) vs. normal birth weight * Sex	1.43	0.74, 2.77	0.29
Sex (women)	1.05	0.84, 1.32	0.67
Age (5 years)	1.21	1.06, 1.18	** < 0.001**
Living with partner	0.73	0.58, 0.92	**0.007**
Socioeconomic status	0.96	0.94, 0.99	**0.002**
**Health behavior**			
Obesity	1.09	0.87, 1.36	0.46
Smoking	1.37	1.08, 1.72	**0.009**
Active sports	0.94	0.77, 1.15	0.55
Alcohol abuse	1.65	0.94, 2.75	**0.007**
**Somatic illness**			
Diabetes	0.57	0.37, 0.87	**0.001**
Cardiovascular disease	1.31	0.93, 1.81	0.12
Cancer	0.87	0.60, 1.24	0.46
COPD/asthma	0.64	0.40, 0.98	0.050
**Psychological comorbidities**			
Anxiety symptoms	1.54	1.14, 2.06	**0.005**
Depression symptoms	11.68	9.05, 15.08	** < 0.001**
Social phobia symptoms	1.93	1.46, 2.53	** < 0.001**
Panic symptoms	1.73	1.26, 2.37	** < 0.001**

COPD, chronic obstructive pulmonary disease; Age, effect per 5 years of age.

LBW was positively related to reports of suicidal ideation (OR 2.84, 95% CI 1.56–4.92). We also observed an interaction with sex (OR 0.48, 95% CI 0.23–1.01), indicating that LBW had different associations with suicidal ideation in men and women (see details below). Participants’ sex alone had no effect. Additional factors related to suicidal ideation were mental distress symptoms (anxiety, depression, social phobia, panic), higher age, smoking and alcohol abuse. Factors negatively associated with suicidal ideation included higher SES, having a partner, and diabetes. High BW was not related to suicidal ideation, and there were no significant interactions of LBW or high BW and sex.

### Associations of suicidal ideation with LBW and other predictors by sex

**Figure 1:**
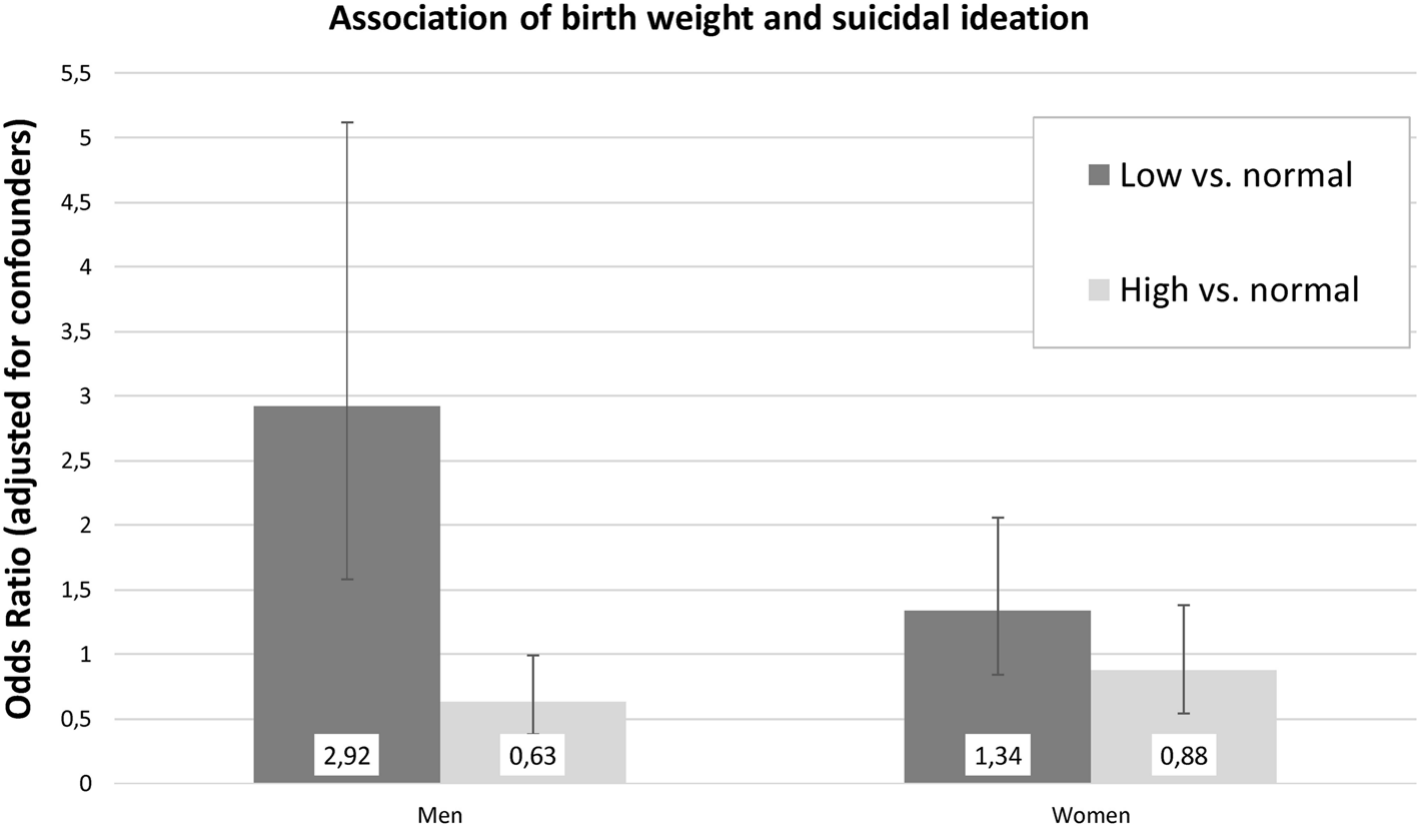
Odds Ratios (incl. 95% CI) for the associations of extreme birth weight categories with suicidal ideation by sex. In men, low birth weight was linked to an almost three-fold increase in reports of suicidal ideation (*p* < .001). In women, the association of low birth weight and suicidal ideation did not reach statistical significance (*p* = .20).

Table 4 shows results of the sex-specific analyses containing all the above mentioned potential predictors (except the interaction terms of sex and BW categories). In men, reports of suicidal ideation were associated with LBW (OR 2.92, 95% CI 1.58–5.12) and with other mental distress symptoms (anxiety, depression, social phobia, panic). Negative associations with suicidal ideation pertained to living in a partnership and diabetes. In women, LBW was not linked to suicidal ideation (*p* = 0.20). Relevant predictors were, as in the male sample, symptoms of anxiety, depression, social phobia, and panic. In women, the likelihood to report suicidal ideation increased with age, lower SES, and smoking. The sex-dependent associations of LBW and suicidal ideation are depicted in Fig. 1.

**Table 4:** Logistic regression of suicidal ideation on birth weight categories controlling for health behaviour, somatic illnesses, and psychological comorbidities (by sex).

	Men (*N* = 3,759, 208 events)			Women (*N* = 4,241, 328 events)		
	OR	95% CI (L, U)	*p*	OR	95% CI (L, U)	*p*
**Demographics**						
Low birth weight (< 2,500 g) vs. normal birth weight	2.92	1.58, 5.12	** < 0.001**	1.34	0.84, 2.06	0.20
High birth weight (> 4,000 g) vs. normal birth weight	0.63	0.38, 0.99	0.06	0.88	0.54, 1.38	0.59
Age (5 years)	1.08	0.99, 1.18	0.08	1.14	1.07, 1.22	** < 0.001**
Living with partner	0.65	0.45, 0.96	**0.03**	0.79	0.60, 1.06	0.12
Socioeconomic status	0.98	0.94, 1.01	0.21	0.95	0.92, 0.99	**0.005**
**Health behavior**						
Obesity	1.06	0.73, 1.51	0.77	1.10	0.82, 1.39	0.59
Smoking	1.39	0.96, 1.98	0.08	1.38	1.02, 1.87	**0.04**
Active sports	0.76	0.55, 1.06	0.11	1.07	0.83, 1.39	0.59
Alcohol abuse	1.87	0.91, 3.53	0.07	1.31	0.46, 3.12	0.58
**Somatic illness**						
Diabetes	0.46	0.23, 0.87	**0.02**	0.70	0.39, 1.20	0.21
Cardiovascular disease	1.35	0.82, 2.16	0.23	1.29	0.79, 2.05	0.29
Cancer	1.33	0.73, 2.31	0.32	0.70	0.43, 1.09	0.13
COPD/asthma	0.45	0.18, 1.01	0.07	0.76	0.44, 1.26	0.30
**Psychological comorbidities**						
Anxiety symptoms	1.78	1.06, 2.93	**0.03**	1.46	1.01, 2.09	**0.04**
Depression symptoms	13.62	8.96, 20.67	** < 0.001**	10.76	7.80, 14.88	** < 0.001**
Social phobia symptoms	1.97	1.21, 3.14	**0.005**	1.83	1.30, 2.56	** < 0.001**
Panic symptoms	1.93	1.07, 3.38	**0.03**	1.67	1.13, 2.43	**0.009**

COPD, chronic obstructive pulmonary disease; Age, effect per 5 years of age.

### Non-responder analysis

In order to address the missing information on BW with respect to 44% of the overall study sample, we conducted a non-responder analysis. The results of the comparison of non-responders and responders on the birth weight item are depicted in Table 5. Responders were younger, more likely to be female, in better physical health, and more likely to report physical activity. They were less likely to report suicidal ideation, but with regard to other mental distress symptoms and health behaviors the group differences were very small.

**Table 5:** Non-responder analysis (*N* = 15,010).

	Responders (*N* = 8,369)	Non-responders (*N* = 6,588)	*p*	*d*
**Demographics**				
Sex (% women)	53.5 (4,474)	44.5 (2,933)	** < 0.001**	0.18
Age (years)	51.5 (10.6)	59.4 (10.1)	** < 0.001**	0.76
Living with partner (%)	81.3 (6,808)	80.9 (5,316)	0.45	0.02
Socioeconomic status	13.59 (4.30)	11.98 (4.55)	** < 0.001**	0.36
**Health behavior**				
Smoking (%)	20.4 (1,710)	18.2 (1,192)	** < 0.001**	0.06
Obesity (%)	23.7 (1,984)	27.0 (1,775)	** < 0.001**	0.07
Active sports (%)	52.8 (4,420)	42.0 (2,769)	** < 0.001**	0.22
Alcohol (gram/day)	4.61 (0/15.71)	4.40 (0/18.65)	**0.01**	0.06
**Somatic illness**				
Diabetes (%)	6.5 (540)	12.9 (847)	** < 0.001**	0.22
Cardiovascular disease (%)	8.5 (709)	15.9 (1,048)	** < 0.001**	0.23
Cancer (%)	7.8 (649)	10.7 (705)	** < 0.001**	0.10
COPD/asthma (%)	4.8 (401)	5.2 (344)	0.23	0.02
**Mental distress (%)**				
Suicidal ideation	6.9 (571)	8.7 (547)	** < 0.001**	0.06
Depression symptoms	7.7 (636)	8.3 (527)	0.18	0.01
Anxiety symptoms	6.8 (560)	6.4 (404)	0.38	0.02
Social phobia symptoms	7.9 (650)	6.7 (427)	**0.01**	0.05
Panic symptoms	4.8 (393)	4.7 (289)	0.69	0.01

Responders: Study participants who disclosed their birth weight. Inference tests between groups (normal/low birth weight) were calculated with t-tests or Chi^2^ tests (where appropriate).

COPD, chronic obstructive pulmonary disease.

## Discussion

We used a large, representative population sample in order to ascertain the associations of low birth weight and suicidal ideation in middle and late adulthood. We aimed to add to previous research on this major public health concern by investigating suicidal ideation in a biopsychosocial context as well as in a sex-sensitive manner. To this end, we calculated multivariate regression models which tested the statistical relevance of LBW alongside other factors such as somatic aspects and health behavior.

Irrespective of their birth weight, 6.8% of the total sample reported suicidal ideation at least on several days over the last two weeks. Compared to other studies which place the 12-month prevalence of suicidal ideation in high-income countries at 2%, this percentage appears quite high. However, one should bear in mind that the respective PHQ-9 item also asks for passive suicidal ideation or death wishes, aspects of suicidality which have been found to be more prevalent among older individuals^1^.

Even after adjusting for age, socioeconomic status, partnership, health behavior, and somatic illnesses, LBW was related to reports of suicidal ideation. This finding is in line with previous research which had attested to detrimental mental health sequelae of LBW in smaller samples with younger participants^17,20^. Importantly, the present results also complement previous work within the context of large cohort studies which has supported associations of low birth weight and suicidal behavior, including deaths by suicide, in younger individuals^5,25^.

In our sample, men born with LBW were almost three times more likely to report suicidal ideation than men with higher birth weight. Previous research has stressed that being born with LBW can be an indicator of intrauterine growth retardation due to a poor nutritional supply during fetal life. This could alter the activity of the hypothalamic–pituitary–adrenal axis and thus predispose an individual to metabolic disease and emotional dysregulation later in life (the thrifty phenotype hypothesis)^41^. Correspondingly, a disturbed neuroendocrine response has been linked to negative physical health consequences including cardiovascular disease and diabetes, and evidence suggests similar mechanisms could mediate the risk for depression in adulthood^7,10^. However, sex-dependent differences regarding HPA axis functioning, its vulnerability for aberrations in the context of early life stress, and outcomes over the life span remain inconclusive^42–44^.

LBW might be a more severe risk factor in men as male babies tend to be born with higher weights, thus falling short of 2,500 g birth weight might implicate more developmental impairment for male babies than for female babies^45^. There have been increased rates of death and need for assistance in very LBW male compared to female infants^46^. These statistics yield important contextual information about the risks associated with being a male LBW baby, especially several decades ago. Importantly, parents of LBW babies were at an increased risk for mental distress symptoms^47^, and interactions of mothers and their preterm infants differed from those of mothers with offspring who had been born to term^48^. In a Canadian study of extremely low birth weight survivors, overprotective parental behavior was related to the presence of anxiety, alcohol and substance abuse disorders in adulthood^49^. Therefore, social moderators or mediators of the effects of somatic vulnerability on (mental) health over the life span need to be considered. This includes social relationships later in life: Adults who were born prematurely/with low birth weight were less likely to attain psychosexual milestones such as having sexual intercourse, a romantic partnership, or to become parents^13^. Thus, they might also experience less communion and other positive effects of close relationships than their peers. A prospective study from the South of Germany showed that the level of social support mediated the association of having been born prematurely or with low birth weight and psychological distress in young adulthood^50^. In our investigation, living together with a partner was associated with fewer reports of suicidal ideation (in men), yet LBW individuals were just as likely as those born with higher BW to live with a partner. However, we did not quantify the extent of support participants received from their partner, or their satisfaction with the relationship. Thus, future research assessing subjective aspects of social ties (instead of structural indicators of social integration) could yield more insight into LBW individuals’ social connectedness.

Further, previous research has suggested long-term detrimental outcomes of LBW with respect to income, educational attainment, and SES^6^. We also observed associations of LBW with lower SES. However, it is important to note that in our study, the direction of effects was not known. The relationship between LBW and SES could potentially be bidirectional. Low SES in mothers is a known risk factor for having a LBW baby^5,51^. From a prevention perspective, targeted support for expectant mothers could address lifestyle factors such as smoking^52^ as they have been found to be the most important determinants underlying the observed effect of lower SES on perinatal outcomes.

The observation that the different kinds of distress all explained statistically significant proportions of the outcome’s variance supports the notion that mental distress might cumulate. Thus, alleviating distress symptoms, especially symptoms of depression, could help counteract suicidal developments.

Sex-specific regression analyses suggested that different factors, including SES and smoking, might be more relevant for women whereas others played a more important role for men’s risk for suicidal ideation. Likewise, previous research has found closer links between smoking and negative affect in women^53^. Whereas age had protective effects for women, men benefitted from living with a partner, suggesting that sex differences also pertain to the sociodemographic context. Interestingly, diabetes was associated with lower risk of suicidal ideation in men. This finding could mirror previous reports of increased (mental) health awareness among men with somatic illnesses. On the other hand, it could also be a methodological artefact as diabetic men have been attested higher rates of mental distress^54^, and a variety of respective symptoms were controlled for in the multivariate model. In fact, we did not observe lower rates of diabetes in participants with suicidal ideation (6.5% vs. 6.4% in those without suicidal ideation). Thus, the importance of this statistical effect should not be overestimated.

Lastly, our results caution against the interpretation of vulnerability and resilience factors derived from investigations which do not employ sex-sensitive methods: Sex-specific analyses revealed that in some cases, effects of predictors observed in the overall sample only applied to men or women. This has implications for research and practice: First, there is a need to adopt research designs and methods of analyses which are sensitive to sex differences^55^, and second, implementation of prevention and intervention strategies which explicitly target individual differences need to take sex into account.

Strong points of the present study are its standardized population-based design, the long follow-up time, and the broad and detailed assessment of sociodemographic factors, psychosocial aspects, mental distress symptoms, health behavioral aspects, and somatic comorbidities. To our knowledge, it is the first representative study in Germany evaluating the association of LBW and suicidal ideation in adulthood.

Limitations pertain to missing data. As about 44% of participants failed to report their birth weight we performed the reported non-responder analysis. As non-responders were older and reported more somatic illnesses, the link between LBW and somatic illnesses may have been underestimated. This might also be the case for associations of LBW and other factors with suicidal ideation as non-responders were more likely to report suicidal ideation. There were also more women in the responder group. Therefore the study’s results might be more representative of their situation. In any case, more research is needed to replicate the observed associations in other large samples and with higher response rates (among men). The above mentioned excess mortality of male LBW babies also highlights the specificity of the present sample: Naturally, long follow up-times (i.e. from birth to middle or late adulthood) introduce a survival bias. Further, information on birth weight was based on participants’ self-report. With the study invitation, they were asked to review personal documents and provide this information. Previous investigations have successfully demonstrated the validity of self-reported birth weight^56,57^. In a population-based study of twins^57^, there was a high intraclass correlation (0.91) between register-based birth weight data and self-reported birth weight. The authors report an overall kappa of 0.71. Furthermore, in a sample of participants born between 1935 and 1960, the intraclass correlation of self-reported birth weight assessed using a questionnaire at two measurement points more than 30 years apart was 0.86. Moreover, previous ophthalmological work using the same cohort sample attested to the congruency of the present birth weight data with governmental reports on the proportion of individuals born with LBW^29^. Another limitation is the inability to include gestational age or preterm birth in our analysis, as this was not surveyed. Consequently, we cannot state if birth weight was low, appropriate, or high in relation to gestational age which is probably one of the main confounders within the present study and should be taken into consideration when interpreting the results. We used only one item to measure suicidal ideation. Given the study design, it was not feasible to include longer questionnaires or personal, clinical interviews although the latter methods are considered superior in correctly identifying individuals at risk^58^. Previous research has reported relations of suicidal ideation and suicide mortality^59,60^, the latter also using the PHQ-9 item used in the present study, and the wish to die and overall mortality^61^. This indicates that reports of suicidal thoughts should be taken seriously. However, risk factors for suicidal ideation should not be equated with risk factors for suicidal actions^62^. For an individual’s progression from suicidal ideation to action, other characteristics such as acquired capability seem to play a role^63,64^. Thus, more research is needed to ascertain the relevance of LBW for suicide attempts and suicide deaths. Additionally, we do not know how many individuals who were born as an LBW baby have died of suicide before the present study was conducted.

As technical advances have vastly improved medical care for LBW individuals, their numbers will continue to grow. However, their quality of life in adulthood should be monitored and additional support, e. g. psychological counselling and long-term aftercare, should be available to counteract detrimental late sequelae such as dangerous suicidal ideation. Our results identify potential targets for interventions such as alleviating psychological distress in the community (especially depression symptoms), offering social support to those in need, and fostering adaptive health behaviors. Along these lines, they also suggest that men and women might benefit from different approaches. Further longitudinal research is needed to clarify the ways in which low birth weight affects physical and mental health outcomes, including potential mediators or moderators such as the social context.
